# huSA: a comprehensive database for multi-dimensional resolution of bulk, single cell and spatial transcription profiles in skin diseases

**DOI:** 10.1093/database/baag009

**Published:** 2026-02-20

**Authors:** Meiling Zheng, Bao Qian, Zhi Hu, Xingyu Wei, Ke Sun, Wenjuan Jiang, Changxing Gao, Ming Zhao

**Affiliations:** Hospital for Skin Diseases, Institute of Dermatology, Chinese Academy of Medical Sciences & Peking Union Medical College, Nanjing, 210042, China; Key Laboratory of Basic and Translational Research on Immune-Mediated Skin Diseases, Hospital for Skin Diseases, Institute of Dermatology, Chinese Academy of Medical Sciences & Peking Union Medical College, Nanjing, 210042, China; Hospital for Skin Diseases, Institute of Dermatology, Chinese Academy of Medical Sciences & Peking Union Medical College, Nanjing, 210042, China; Key Laboratory of Basic and Translational Research on Immune-Mediated Skin Diseases, Hospital for Skin Diseases, Institute of Dermatology, Chinese Academy of Medical Sciences & Peking Union Medical College, Nanjing, 210042, China; Hospital for Skin Diseases, Institute of Dermatology, Chinese Academy of Medical Sciences & Peking Union Medical College, Nanjing, 210042, China; Key Laboratory of Basic and Translational Research on Immune-Mediated Skin Diseases, Hospital for Skin Diseases, Institute of Dermatology, Chinese Academy of Medical Sciences & Peking Union Medical College, Nanjing, 210042, China; Hospital for Skin Diseases, Institute of Dermatology, Chinese Academy of Medical Sciences & Peking Union Medical College, Nanjing, 210042, China; Key Laboratory of Basic and Translational Research on Immune-Mediated Skin Diseases, Hospital for Skin Diseases, Institute of Dermatology, Chinese Academy of Medical Sciences & Peking Union Medical College, Nanjing, 210042, China; Hospital for Skin Diseases, Institute of Dermatology, Chinese Academy of Medical Sciences & Peking Union Medical College, Nanjing, 210042, China; Key Laboratory of Basic and Translational Research on Immune-Mediated Skin Diseases, Hospital for Skin Diseases, Institute of Dermatology, Chinese Academy of Medical Sciences & Peking Union Medical College, Nanjing, 210042, China; Hospital for Skin Diseases, Institute of Dermatology, Chinese Academy of Medical Sciences & Peking Union Medical College, Nanjing, 210042, China; Key Laboratory of Basic and Translational Research on Immune-Mediated Skin Diseases, Hospital for Skin Diseases, Institute of Dermatology, Chinese Academy of Medical Sciences & Peking Union Medical College, Nanjing, 210042, China; Hospital for Skin Diseases, Institute of Dermatology, Chinese Academy of Medical Sciences & Peking Union Medical College, Nanjing, 210042, China; Key Laboratory of Basic and Translational Research on Immune-Mediated Skin Diseases, Hospital for Skin Diseases, Institute of Dermatology, Chinese Academy of Medical Sciences & Peking Union Medical College, Nanjing, 210042, China; Hospital for Skin Diseases, Institute of Dermatology, Chinese Academy of Medical Sciences & Peking Union Medical College, Nanjing, 210042, China; Key Laboratory of Basic and Translational Research on Immune-Mediated Skin Diseases, Hospital for Skin Diseases, Institute of Dermatology, Chinese Academy of Medical Sciences & Peking Union Medical College, Nanjing, 210042, China; Department of Dermatology, The Second Xiangya Hospital of Central South University, Changsha, 410011, China

## Abstract

**Background:**

Skin diseases are among the most prevalent conditions worldwide, posing significant threats to human health by causing physical discomfort, psychological distress, and reduced quality of life. With the rapid advancement of high-throughput technologies, a substantial number of transcriptomic datasets, including single-cell RNA sequencing (scRNA-seq), spatial transcriptomics, and bulk RNA-seq, have been generated in the field of dermatology over the past decade. However, the lack of effective integration and standardized analysis pipelines limits the full utilization of these valuable resources in skin disease research.

**Objectives:**

To address this gap, we aimed to construct a comprehensive, integrative, and user-friendly atlas that enables systematic exploration of skin transcriptomic data across multiple diseases and modalities.

**Methods:**

We developed the Human Skin Atlas (huSA) (‘https://humanskinatlas.com/index.html’), a publicly accessible database that incorporates data from 17 skin diseases and 63 independent datasets, including 1 434 scRNA-seq, 63 spatial transcriptomics, and 1 502 bulk RNA-seq samples. The database provides standardized cell-type annotations, differential gene expression analysis, cell-cell interaction mapping, pathway and metabolic module enrichment, transcription factor regulatory inference, and differentiation state assessment for scRNA-seq data. Data from identical skin diseases were further integrated to enhance biological signal detection. For visualization, we embedded the ‘cell × gene’ and ‘Cirrocumulus’ platforms, offering interactive and customizable gene expression visualizations at both single-cell and spatial levels with user-defined parameters.

**Results:**

The huSA enables both individual dataset analysis and cross-dataset integration, providing robust, consistent, and scalable insights into skin disease biology. Demonstration analyses confirmed that results derived from either single datasets or aggregated multi-dataset integrations exhibited high reliability and biological relevance. The platform successfully supports diverse research needs, including cell-type-specific expression profiling, regulatory network construction, and spatial transcriptomic exploration.

**Conclusions:**

The Human Skin Atlas (huSA) represents a state-of-the-art integrative resource for the skin research community. By offering multiscale analyses and interactive visualization tools, the huSA accelerates the discovery of molecular mechanisms underlying skin diseases and facilitates translational research efforts aimed at improving skin health.

## Introduction

The skin, the largest organ of the body and accounting for about 16% of body mass, serves as our first line to defense against toxic substances, solar radiation, and pathogenic threats and forms a protective barrier against the external environment [1–4]. As described in the Global Burden of Disease(GBD) studies, skin diseases such as bacterial skin infections, acne, psoriasis, urticaria and other skin diseases are leading cause of global health burden, making a huge impact on hundreds of millions of people in the world [5–7]. Skin diseases encompass a wide range of conditions, with the most common being inflammatory disorders such as urticaria [8], contact dermatitis [9], and atopic dermatitis [10]. Another common category includes autoimmune skin diseases, such as psoriasis [11,12], systemic lupus erythematosus [13], systemic sclerosis [14], vitiligo [15] and pemphigus [16]. These skin conditions are characterized by an abnormal immune response, in which the immune system fails to recognize the body’s own tissues and erroneously targets the skin even other organs such as kidney [17].

Although genetic, environmental, and other factors contribute to immune dysregulation and metabolic disturbances, which play a crucial role in the pathogenesis of skin diseases [18,19], the underlying mechanisms of many skin diseases remain poorly understood. By the way, traditional therapeutic agents, such as corticosteroids and immunosuppressants, are known to have significant toxic side effects, while targeted therapies face the challenge of non-responsiveness in some patients [14,20]. Additionally, 58% of psoriasis patients experience relapse annually, the 7-year relapse rate of atopic dermatitis patients is as high as 75.9% [10], and approximately 60% of systemic lupus erythematosus patients have disease persistent or recurrent, making it difficult to achieve a cure [21]. Furthermore, many skin diseases are associated with comorbidities. Approximately 30% of psoriasis patients develop psoriatic arthritis [22], which can lead to disability. Around 16.7% and 33.7% of atopic dermatitis patients also suffer from asthma and allergic rhinoconjunctivitis, respectively [23]. Patients with systemic lupus erythematosus often experience multi-organ involvement, and their risk of premature death is higher than that of the general population, with an average life expectancy shortened by ten of years [24]. Recurrent disease flare-ups and life-threatening comorbidities present significant challenges in the diagnosis and treatment of skin diseases. Thus, more in-depth studies are required to better understand the underlying immunological mechanisms and to develop targeted therapies for patients with skin diseases including autoimmune skin diseases [25,26].

Despite these challenges, advancements in high-throughput sequencing technology have significantly advanced our understanding of pathogenesis in skin diseases, facilitated the discovery of disease biomarkers, and contributed to the development of new therapy. Single-cell RNA sequencing (scRNA-seq), first proposed by Tang et al. in 2009, has become one of the most widely used transcriptomics methods of the last decade [27]. High-throughput technology such as RNA sequencing and single-cell sequencing help to construct of individualized biological networks and are employed to address skin tissue complexity and cellular heterogeneity. Normal-appearing skin exhibits a type I interferon-rich environment that primes immune and skin cells towards proinflammatory states in cutaneous lupus erythematosus [28]. Immune cells such as T, B and NK cells enriched more frequency in cutaneous lesions of DLE than those of SLE [29]. A unique subset of age-associated T helper cells (ThA) plays a dual role in cytotoxic and B cell helper functions, contributing to increased disease activity in SLE [30]. A novel population of COL6A5^+^COL18A1^+^ fibroblasts secretes CCL2 and CCL19 cytokines, which recruit LAMP3^+^ dendritic cells expressing CCL19 and CCR7, thereby driving type 2 inflammatory responses in AD lesions [31]. The SFRP2^+^ fibroblasts amplify the inflammatory response by releasing cathepsin S, which in turn activates IL36G expression in keratinocytes of psoriasis [32]. These studies significantly enhanced our understanding of skin diseases and proposed potential biomarkers and targets for clinical diagnosis and therapies. Despite these advances, different skin sequencing datasets exhibit distinct quality control measures and inconsistent analysis methods, which limit the generalizability of their results.

With the rapid increase in high-throughput data, a variety of general single-cell databases have been developed, such as the Single Cell Expression Atlas (https://www.ebi.ac.uk/gxa/sc/home) by EMBL-EBI and the Single Cell Portal (https://singlecell.broadinstitute.org/single_cell) by the Broad Institute. Many specific single-cell databases have also been developed, such as the cancerSCEM [33] (https://ngdc.cncb.ac.cn/cancerscem/index) by China National Center for Bioinformation, SCAR [34], ABC portal [35]. However, many freely available datasets are missing cell-type annotations, hindering the efficient repurposing of published skin disease-related data. Meanwhile, most of these datasets focus on a single skin disease and lack comparative analysis across multiple affected tissues. Moreover, a comprehensive interactive database of high-throughput bulk and single-cell transcriptomes specific to skin diseases is currently unavailable. Thus, there is an urgent need for a specialized database that focus attention on the skin field and provide separate and integrate analysis for various skin conditions.

To meet the growing demand for an intensive, integrated and standard data analysis database in skin diseases, we represent a comprehensive database merged muti-dimensional datasets for skin diseases, named human skin atlas (huSA, https://humanskinatlas.com/index.html). This database integrates bulk, single-cell, and spatial transcriptomics data from various skin diseases which not only involve tissues such as skin lesions but also other affected tissues like blood, kidney and synovium, et al. The huSA version 1.0 encompasses 4 653 696 high-quality single cells from 1434 scRNA-seq samples covering 17 skin diseases, along with 1502 bulk RNA-seq samples and 63 spatial transcriptions samples. All the single cell sequencing datasets offer a unified analysis pipeline, including cell clustering, cell annotation, identification of differentially expressed genes, pseudotime trajectory inference, cell-cell interaction analysis and cell-type specific regulon calculation. Furthermore, we also furnish users with online analysis on different datasets and provide integrated data analysis for some skin diseases, which significantly enhances the reliability and comprehensiveness of the results. The verification of single-dataset or integrative analysis of multiple datasets further validate the reliability and applicability of the huSA. In short, we anticipate that huSA foster a user-centric environment which contributes to data visualization and data mining on skin diseases.

## Materials and methods

### Data collection

Literatures related to single cell, bulk RNA sequencing, spatial transcriptome sequencing and skin diseases in PubMed were manually confirmed whether their datasets were publicly available. In addition, we searched and download accessible datasets from Gene Expression Omnibus (https://www.ncbi.nlm.nih.gov/geo/), ArrayExpress (https://www.ebi.ac.uk/arrayexpress/) and Zenodo (https://zenodo.org/) to make our database more comprehensive. By manually reviewing supplementary materials in the references, we compiled the metadata for each dataset, including details such as disease type, technology used, tissue origin, sampling site, and interference. In single-cell sequencing, the raw sequencing datasets and expression matrices accounted for 62.69% and 37.31%, respectively. In summary, the database comprises 61 datasets, including scRNA-seq, bulk RNA-seq, and spatial transcriptomics data from 17 different skin disease types.

### scRNA-seq data processing

We developed a standardized set of pipelines to manage these data. Firstly, scRNA-seq data in SRA or Bam format were converted to FASTQ format using either parallel-fastq-dump v0.6.7 (https://github.com/rvalieris/parallel-fastq-dump) or bamtofastq v1.4.1 (https://github.com/10XGenomics/bamtofastq), respectively. Next, Cell Ranger v8.0.0 [36] software (https://www.10xgenomics.com/cn/support/software/cell-ranger/downloads) was employed to generate a unique molecular identifier (UMI) count matrix for each sample, utilizing its built-in reference data (Human reference: GRCh38-2024-A). Counts or normalized data were included for the rest of the scRNA-seq data. The following quality control criteria were used to select cells and genes in high grade: (1) nFeature_RNA greater than 200, (2) mitochondrial percentage less than 10%, (3) cells with more than 200 genes, and (4) genes detected in at least three cells. The Scrublet package was used to assess and subsequently eliminate doublets in all datasets [37]. Afterwards, the downstream analysis was conducted by means of a standard python workflow [38]. Briefly, we used the scanpy package v1.10.1 (https://github.com/scverse/scanpy) to perform a succession of analyses, which included normalization, selection of highly variable genes, scaling of the processed data, dimension reduction through principal component analysis (PCA), de-batching with Harmony, cell clustering with appropriate number of principal components and clustering resolutions, and visualizing using uniform manifold approximation and projection (UMAP). Moreover, we ensured that the original cell clustering and annotation were retained whenever metadata was accessible. Since there was no cell annotation data in the original paper, we assigned cell labels to each cluster according to the unique expression of canonical cell markers. To improve the precision and efficiency of cell annotation, we additionally incorporated machine annotations results from CellTypist and scMayoMap [39,40].

### Transcription factors and cell-cell interactions analysis

The pySCENIC (version 0.12.1) was used to assess the enrichment of transcription factors and the activity of regulators in each sample [41]. Briefly, the ‘pyscenic grn’ command was used to infer the transcription factor co-expression modules from the single-cell expression matrix. Subsequently, the ‘pyscenic ctx’ function analyzed transcription factor motif enrichment to identify direct targets. Finally, the ‘pyscenic aucell’ command assessed the enrichment of regulons within individual cell type. To identify master regulators, we employed two approaches: the Regulon Specificity Score (RSS) method using the calcRSS function in the SCENIC R package (version 1.3.1), and the evaluation of average regulon activity within each group. CellPhoneDB (version 5.0.1) [42] was utilized to predict intercellular communication between distinct cell types within each sample in the cell interaction submodule, with the parameters set to ‘-iterations = 1000 -threshold 0.01 -pvalue 0.05 -subsampling false -subsampling-log false -subsampling-num-cells 1000’. The results were then visualized using the OmicVerse python package (version 1.6.3) [43].

### Inference of cell developmental trajectories

The trajectories analysis was developed to predict cell differentiation states using CytoTRACE2 [44], a tools within the Omicverse package. CytoTRACE2 was employed to calculate the differentiation scores for each cell type under various conditions, such as disease and healthy states, allowing for a comparison of developmental potential across these states. The scores range from 0 to 1, where a higher score denotes greater stemness (less differentiation) and a lower score suggests a more differentiated state. Additionally, we identified differentially expressed genes for each cluster within each cell type, which revealed distinct gene expression patterns corresponding to different levels of differentiation.

### Calculation of cell type specific metabolic activity

We took advantage of highly-advanced flux estimation tool, scFEA [45] (version 1.1.2) for calculating cell type specific metabolic activity. This remarkable toll encompasses a comprehensive set of 168 metabolic modules and 70 pathways. We formatted the data according to the structure and input specifications required by the scFEA package. Leveraging its unique algorithm and metabolic module/pathway architecture, scFEA inferred the metabolic flux for each individual cell and mapped single-cell gene expression data onto the relevant metabolic modules and pathways. This mapping enabled gene expression levels to be linked with specific metabolic functions and processes.

### Integration datasets

To fully leverage the value of existing data and improve the reliability of results, we integrated data, and provided analysis results from the same disease, from skin or blood. Given that different dataset used different reference genomes during the processing by Cell Ranger, we employed Ensembl Gene ID for data integration instead of gene symbols. Subsequently, we mapped the Ensembl Gene ID back to gene symbols in accordance with the GTF file of the GENCODE v44 version. We computed the pearson correlation of gene expression within a particular cell type of certain diseases, which can be utilized to deduce genes with analogous expression patterns. Other analysis are mentioned above.

### Analysis of spatial and bulk transcriptomic dataset

Spatial transcriptomics datasets were analyzed through the Omicverse package. Specifically, the ‘ov.pp.preprocess’ function was adopted to process the gene expression matrix. Spatial domain recognition was then performed using GraphST, and the spatial data was clustered by the mclust, leiden, or louvain algorithms function [46–48]. The bulk RNA sequencing datasets were analyzed by the RNAseqStat2 package (https://github.com/xiayh17/RNAseqStat2). The RNA-seq analysis included the followings: differentially expressed genes (DEGs), Gene Ontology (GO), Kyoto Encyclopedia of Genes and Genomes (KEGG), Gene set enrichment analysis (GSEA), and Gene Set Variation Analysis (GSVA).

### Distribution of fibroblast clusters across disease phenotypes

The proportional representation of different diseases within the total fibroblast population was calculated by the Ratio of observed to expected (Ro/e) cell numbers. For this analysis, expected cell numbers for each fibroblast cluster were derived from chi-square tests [49]. To investigated alterations in fibroblast subpopulation abundance in prurigo nodularis (PN) relative to healthy controls (HC). A k-nearest neighbour (kNN) graph was constructed based on the integrated dataset of fibroblasts from PN and HC samples. Differential neighbourhood abundance testing was performed using MiloR (accessed via the pertpy toolkit, Version 0.10.0) with default settings [50].

### cNMF analysis

We applied consensus Non-negative Matrix Factorization (cNMF) [51] to raw gene expression counts from PN samples to uncover distinct gene programs. Adhering to cNMF’s recommended procedures, our analysis included the top 2 000 highly variable genes and involved 100 factorization iterations, while other settings remained at their defaults. The critical hyperparameter K, representing the number of gene programs, was determined by testing a range from 20 to 50 in steps of 1. We selected K = 43 as it offered the most favorable compromise between factor stability and overall model error. To refine the consensus gene programs, approximately 5% of fitted cNMF spectra were filtered out if their mean distance to k-nearest neighbors surpassed 0.3.

### Database implementation

The huSA database was built using Docker, Redis, Nginx, Spring Boot, Bootstrap, MySQL, and MinIO as its primary components and frameworks. The backend system was developed in Spring Boot, with the web service built and deployed using Maven. Bioinformatics analysis was facilitated through the containerized deployment of R-related applications. The frontend leverages HTML5, CSS, AJAX, jQuery, and Bootstrap were used for rendering and interactive operations. ECharts, BootstrapTable, Chart.js, and Plotly.js were applied to create interactive interfaces and visualizations. Interaction between the frontend and backend was managed through REST APIs, ensuring scalability and compatibility. The database utilized MySQL as the primary data storage solution, with MinIO serving as the file storage container. MyBatis was employed as the data access layer, ensuring security and persistence of stored data. The Google Chrome (v56.0 and above), 0pera (v53.0 and above), Safari (vi.1 and above) or Firefox (v64.0 and above) are recommend to achieve a better user experience.

## Results

### Overview of huSA

The huSA is a public database designed to analyze, visualize, and integrate bulk RNA-seq, single-cell RNA-seq, and spatial transcriptome data related to human skin diseases. The available datasets were retrieved from GEO (NCBI), GSA (NGDC), ArrayExpress (EBI), and ZENODO. The database facilitates detailed exploration of cellular characteristics and gene expression dynamics in skin diseases. All single-cell sequencing datasets provide a standardized analysis pipeline, which includes quality control, cell clustering, cell annotation, differentially gene expression analysis, cell type or subtype proportion quantification, pseudotime trajectory inference, cell-cell interaction analysis, cell-type-specific regulon calculation and cell-type metabolism analysis. These scRNA-seq datasets were primarily generated using the 10 × Genomics and Singleron platforms. All bulk RNA-seq datasets support analyses such as DEGs, GO, KEGG, GSEA, and GSVA. The database includes standardized sample metadata and analysis results, comprising 1 434 scRNA-seq samples from 38 projects covering healthy control and 17 skin disease types, 63 spatial transcriptomic samples from 5 projects, and 1 502 bulk RNA-seq samples. After aligning to human genomic sequences and conducting cell quality control, 4 653 696 high-quality single cells were retained for downstream analysis. Beyond sequencing data from skin lesions, the database incorporates data from other tissues and organs affected by skin diseases, including blood, lungs, and synovial fluid, allowing for more comprehensive and integrated analyses. The database encompasses various disease types and their respective sample counts, as presented in Supplementary Table 1.

**Figure 1:**
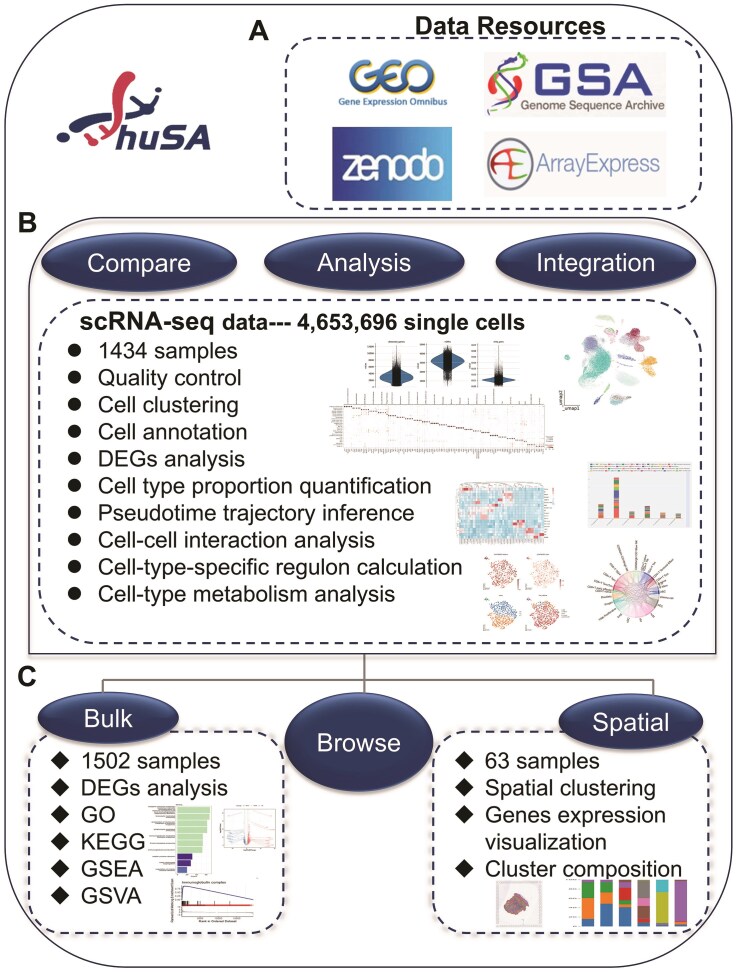
Overview of huSA. All datasets were mainly resourced from 4 database. The scRNA-seq data, bulk RNA-seq data and spatial transcription data were performed a series of analysis. Besides ‘Home’ page, the huSA provides other 6 functional modules, the ‘Compare’, ‘Analysis’, and ‘Integration’ module were used to analysis data from scRNA-seq datasets.

To ensure consistency, unified cell type annotations were assigned to each dataset within the same disease. These standardized annotations, derived from the cell labels used in the integrated dataset, significantly enhance the comparability and reliability of the analytical results within the database. For single-cell datasets of skin tissue, we basically annotated 11 major categories of skin stromal cells and immune cells, along with 53 cell subtypes. Similarly, we named 8 major cell categories and 25 subtypes for single-cell sequencing data of blood samples. Additionally, huSA supports robust and precise cross-dataset comparisons by implementing a standardized analytical methodology, further bolstering the databases utility and reliability. The ‘Home’ page presents an overview of the database workflow and core content, news on current and upcoming updates, as well as citation and contact information. In addition to ‘Home’ page, the huSA offers 6 main functionalities: ‘Browse’, ‘Compare’, ‘Analysis’, ‘Spatial’, ‘Integration’, and ‘Bulk’. The ‘Spatial’ and the ‘Bulk’ functionalities represent the analysis of spatial transcriptome data and bulk RNA-seq data, respectively. The ‘Compare’, ‘Analysis’ and ‘Integration’ pages offer different results of scRNA-seq data (Fig. 1).

### Dataset exploration

**Figure 2:**
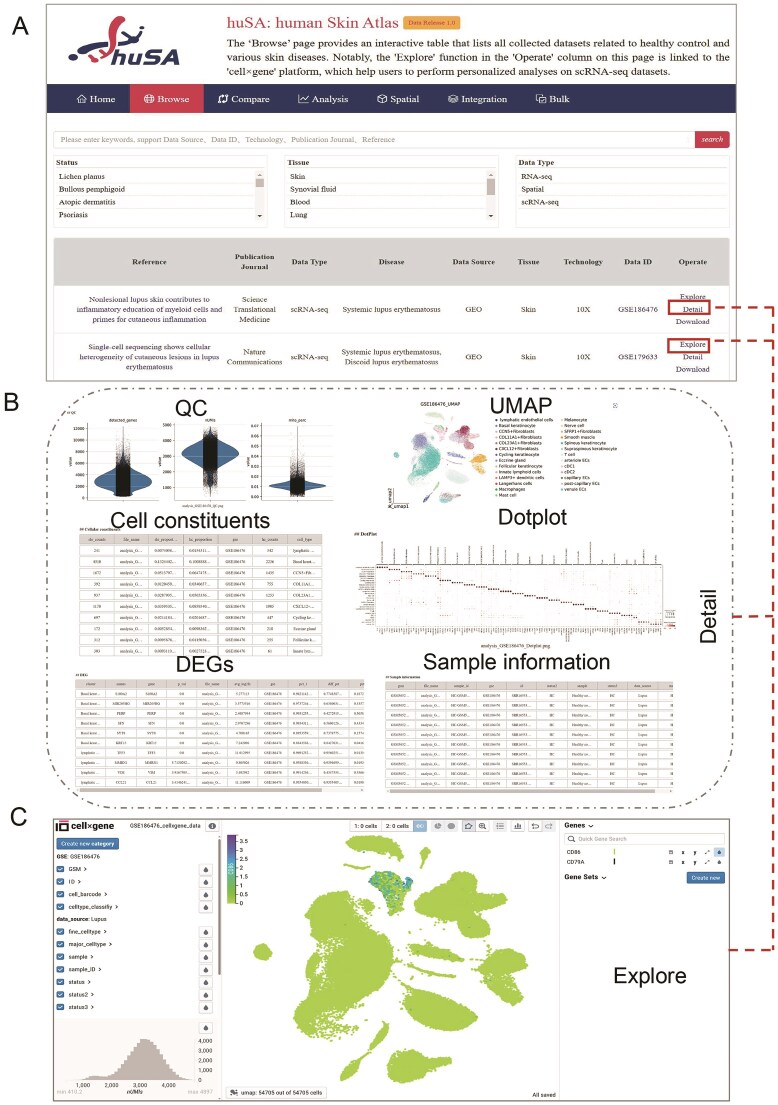
The browse interface of huSA. (A) An overview of the ‘Browse’ pages, including a Table displaying all collected datasets, detailed information, and the search functionality. (B) An additional page demonstrating that the ‘Detail’ option provides users with detailed results of scRNA-seq datasets. (C) The ‘cell × gene’ platform that links to the ‘Explore’ options in the ‘Operate’ column, enabling users to investigate gene expression patterns in scRNA-seq datasets.

One of the key strengths of huSA is its capability to implement dataset-specific filters and combinations, allowing for highly tailored sample analysis. The ‘Browse’ page provides an interactive Table that lists all collected datasets related to healthy control and various skin diseases. This Table provides detailed information such as reference, publication journals, data types, diseases, data sources, tissues, technologies, data ID and operation options. The ‘Reference’ column contains external hyperlinks to the pages of published articles, while the ‘Data ID’ column directs users to the corresponding dataset resource pages. Users can enter keywords of data source, data id, technology, and reference, or select one or more criteria of Status, Tissue and Data Type, to locate their required data (Fig. 2A).

The ‘Detail’ option in the ‘Operate’ section provides hyperlinks directing users to the specific analysis results for each scRNA-seq dataset, while the ‘Detail’ options for RNA-seq and spatial transcriptomic links to the pages of ‘Bulk’ and ‘Spatial’, respectively. These scRNA-seq results include of QC, UMAP visualization, cellular constituents, dotplots of genes expression used for cell annotation, DEGs and sample information (Fig. 2B). The ‘Explore’ in ‘Operate’ column offers users with a seamless and intuitive dataset analysis interface through the ‘cell × gene’ platform. Users can leverage various interactive tools and visualization methods within this feature to investigate gene expression patterns, gaining valuable insights in a straightforward and accessible manner (Fig. 2C). The ‘Download’ function enables users to download ‘h5ad’ files of each dataset, providing the flexibility to perform personalized analysis as needed. Overall, the ‘Browse’ function significantly enhances the ease of exploring and analyzing datasets with huSA, empowering users with robust tools for both standard and customized investigation.

### Dataset comparison

The huSA provides a user-friendly platform for cross-dataset comparisons via the ‘Compare’ module, enabling users to efficiently identify and analyze similarities and differences in cellular and gene-level data across multiple scRNA-seq datasets. The cell comparison function encompasses two primary features: dataset comparison and sample comparison, both of which are designed to provide users with comprehensive tools to analyze and visualize differences in cellular and gene-level data across multiple datasets.

**Figure 3:**
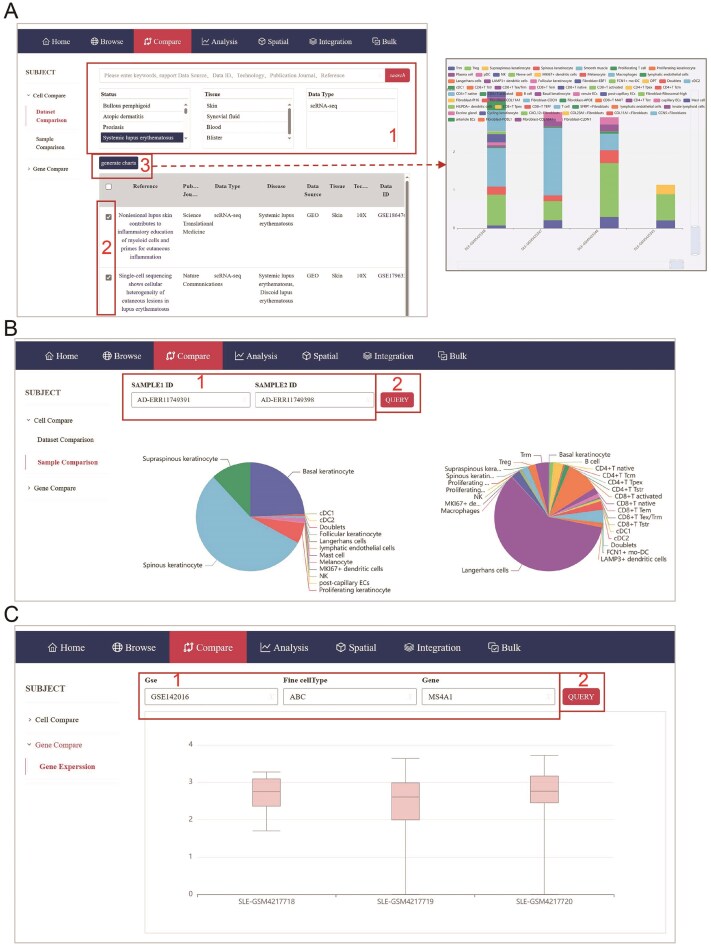
Compare module of huSA database. (A) The page accompanied with red frame indicates step by step administration for dataset comparison. (B) The page accompanied with red frame shows the method of sample comparison. (C) A page displays the gene expression visualization.

In the dataset comparison, in addition to searching with the same keywords as in ‘Browse’ modules, users can refine their search by selecting one or multiple criteria, such as status, tissue, or data type, to filter relevant datasets. After refining their search, users can select specific datasets and click ‘Generate Charts’ to produce visualized results, including detailed histograms (Fig. 3A). The scroll bars located along the rows and columns of the histogram allow users to customize the display by selecting the number of samples or cell types they wish to visualize, ensuring clarity and flexibility in data presentation. In the sample comparison, after selecting sample IDs, the system generates an interactive pie chart that visualizes the proportion of each cell type in the chosen samples, providing an intuitive overview of cellular composition (Fig. 3B). In the Gene Expression feature of the Gene Compare function, users can compare the expression levels of selected genes in chosen cell types across various samples within selected GSE datasets using bar graphs (Fig. 3C).

### Analysis modules

The ‘Analysis’ module offers four interactive and user-friendly analysis options, each designed to address key aspects of single-cell data interpretation. These options provide powerful tools for in-depth exploration and functional analysis of cellular status and cell-cell interactions. The cell-cell interaction option, powered by CellPhoneDB, enables users to investigate and visualize communication networks between different cell types, uncovering key ligand-receptor interactions that drive biological processes. The transcription factor inference module, utilizing pySCENIC, identifies and infers regulatory networks by analyzing transcription factors and their target genes, providing insights into the underlying regulatory mechanisms within cells. The metabolic pathway enrichment analysis module, implemented through scFFA, offers a detailed exploration of metabolic pathways, highlighting pathway activity variations across different cell populations to reveal metabolic shifts associated with disease states or cellular functions. The differentiation potential analysis module, powered by CytoTRACE2, evaluates and predicts the developmental trajectory and differentiation potential of cells, helping users to understand lineage relationships and developmental hierarchies within their datasets.

**Figure 4:**
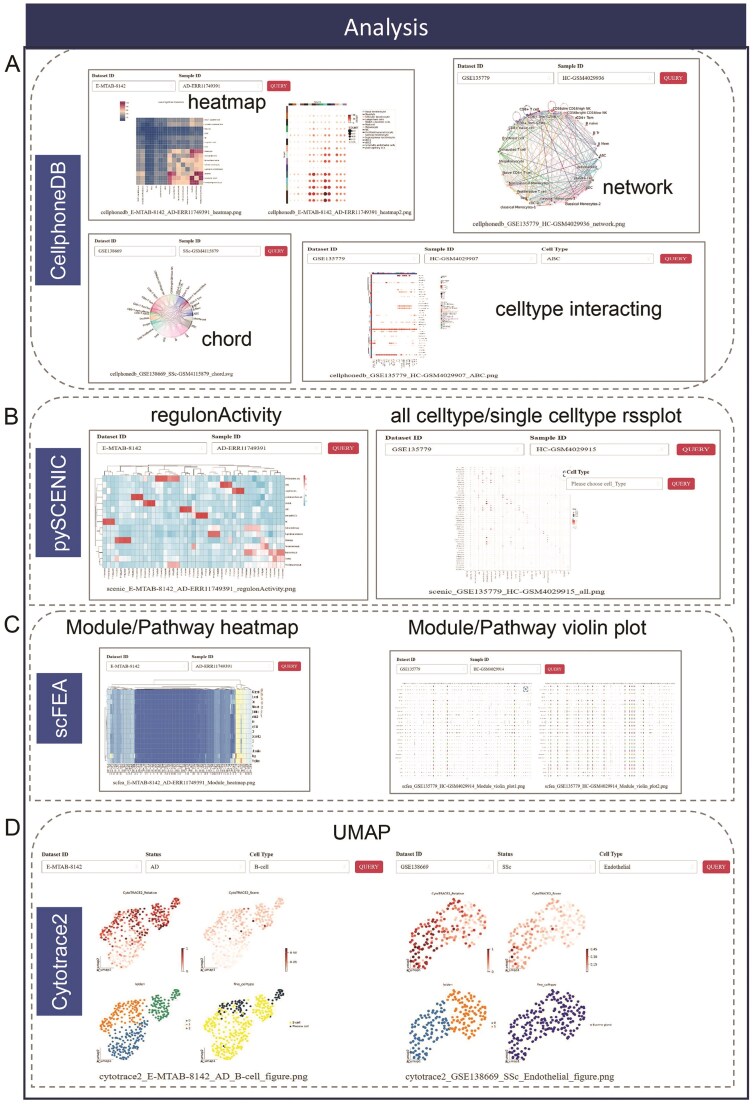
Four main advanced function in the ‘Analysis’ module. (A) The CellphoneDB includes 4 different visualized method to show the results of cell-cell interaction. (B) The pySCENIC encompasses heatmap to show regulon activity and rssplot to display all cell type/single cell type rss score. (C) Heatmap and violin plot represent enriched metabolic module/pathway in the analysis of scFEA. (D) The UMAP shows the predicted cell status of differentiation.

The CellphoneDB option enables users to visualize interactions between cell types and explore the ligand-receptor network by selecting specific parameters selection such as ‘Dataset ID’, ‘Sample ID’ and ‘Cell Type’. Interaction strengths between cell types are represented in the heatmap item, while the chord and network items represent interactions with interaction pairs count exceeding 15. Additionally, the ‘celltype interacting’ item offers a detailed representation of the concrete ligand-receptor pairs for interaction between selected cell type and other cell types (Fig. 4A). All results can be magnified through left mouse click. With the query system of pySCENIC, users can examine transcription factors regulated networks in depth. The item presents the activity of each regulon across various cell types in the ‘regulonActivity’ tab, while the ‘all celltype rssPlot’ displays the regulon specificity score, the ‘single celltype rssPlot’ highlights the specificity of each regulator’s activity (Fig. 4B). The scFFA inferres metabolic fluxes and cell type dynamic levels. By specifying a dataset ID and sample ID of interest, users can effortlessly generate module/pathway heatmaps and violin plots that display enriched metabolic modules and pathways (Fig. 4C). All analysis results are readily exportable; users can simply right-click and select the ‘Save as Picture’ option to download the results locally. After applying hierarchical filtering, the module emphasizes four primary ‘UMAP’ tab within the Cytotrace2, which illustrates the predicted differentiation states and their corresponding cell types (Fig. 4D). Additionally, the ‘DEG’ tab offers detailed DEGs across selected cell type, providing valuable insights into cellular differentiation and gene expression patterns. Together, these items provide a comprehensive suite of tools for advanced single-cell analysis, enabling researchers to uncover complex biological insights with precision and ease.

### Integration, spatial and bulk modules

**Figure 5:**
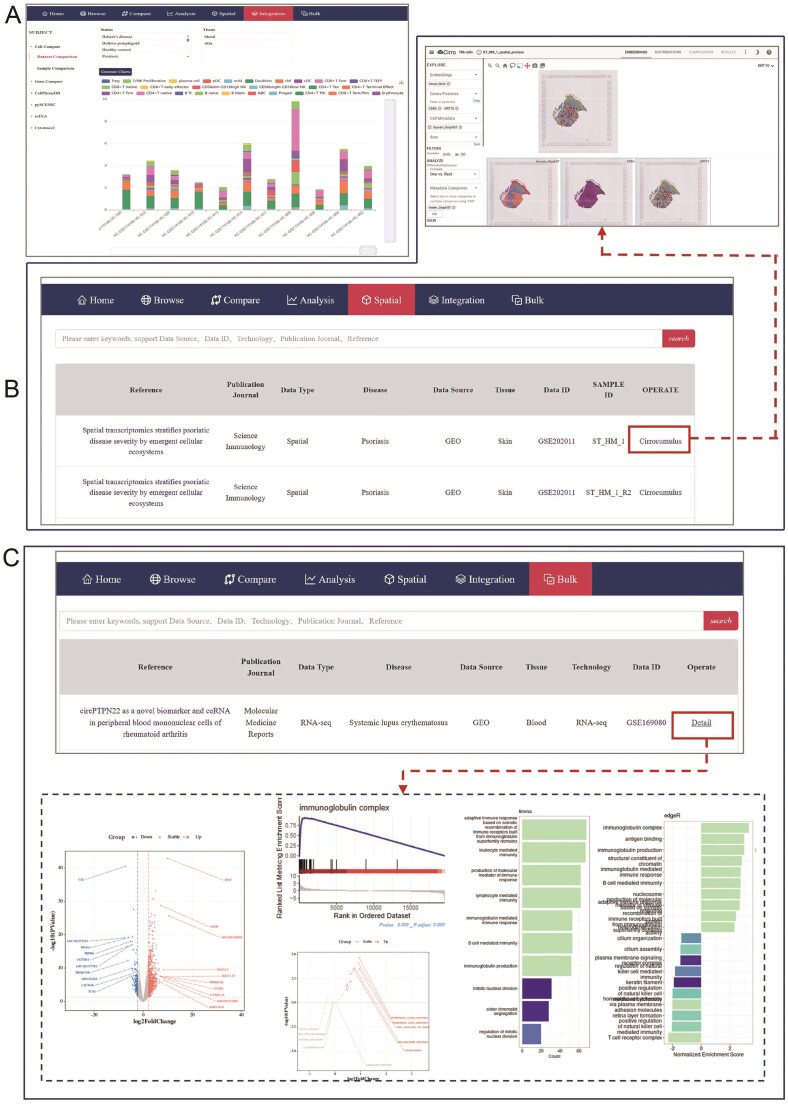
The pages of ‘Integration’, ‘Spatial’ and ‘Bulk’ modules. (A) The page shows 17 functions options of Integration. (B) The website displays the messages of collected spatial dataset, the red box and red line refers to the ‘Cirrocumulus’ platform which is equipped for the personized visualization of spatial transcription data. (C) The page represents settings of ‘Bulk’ module, the red box and red line refers to the ‘Detail’ items which links to a variety analysis of individual bulk RNA-seq data.

The application of this ‘Integration’ module is consistent with the ‘Compare’ and the ‘Analysis’ module previously discussed, but it utilizes aggregated data to enhance its utility (Fig. 5A). This module provides unified datasets and standardized analysis results for the same disease, enabling researchers to delve into biological insights with the advantage of larger, more comprehensive datasets. This module not only facilitates deeper cross-dataset comparisons but also empowers users to uncover complex patterns and relationships that might otherwise remain undetected in smaller, isolated datasets.

The ‘Spatial’ and ‘Bulk’ encourage users to derive deeper biological insights by leveraging data from different levels of transcription datasets. The ‘Spatial’ module functions similarly to the Browser module, but the ‘Operate’ columns direct users to the ‘Cirrocumulus’ platform. This platform offers an intuitive interface for spatial transcriptomics data analysis. Users can choose different types of cell metadata to explore gene expression patterns and spatial location information across different samples using a variety of functions available on the platform (Fig. 5B). The ‘Bulk’ module incorporates bulk RNA-seq datasets, which provide superior sequencing depth and coverage compared to single-cell sequencing. This module features a Table listing all collected bulk RNA-seq projects related to various skin diseases. The ‘Operate’ in the Table are hyperlinked, leading to detailed information on the datasets and comprehensive analysis such as DEGs, GO, KEGG, GSEA for each dataset (Fig. 5C). Together, these modules enhance the scope of data exploration and facilitate robust transcriptomic analysis across multiple scales.

### Comprehensive analysis of single-cell datasets by huSA reveals the pathophysiology of psoriasis

**Figure 6:**
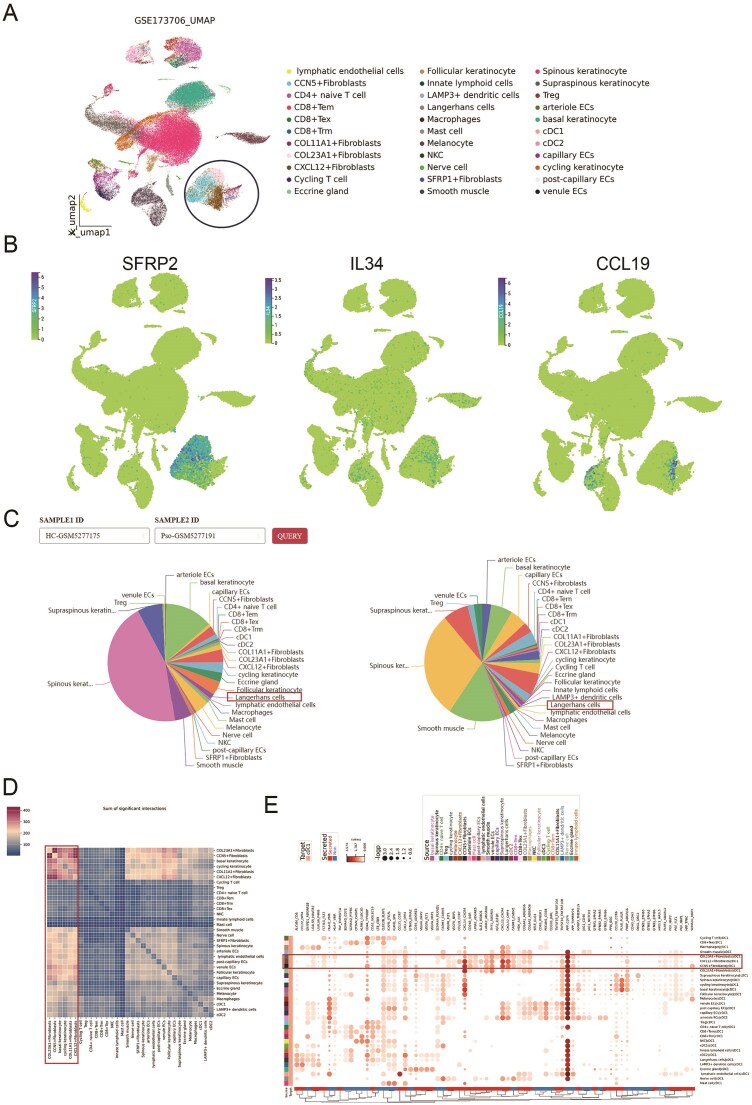
Analysis of scRNA-seq dataset for psoriasis through huSA. A. UMAP plot shows the detailed subtypes of the dataset (GSE173706). B. UMAP plots represent key genes expression on fibroblasts. C. Pie plots display the subtype distribution in the individual sample. D. The heatmap shows the sum of significant interactions among all subtypes. E. The dotplot shows multiple ligand-receptor pairs expression among interactive cell type.

To validate the single-cell data analysis pipeline and features available to huSA users, we selected a psoriasis research dataset (GSE173706) for demonstration. Based on differential gene expression analysis, we identified and annotated six keratinocyte subtypes, five fibroblast subtypes, and five endothelial cell subtypes. In addition, we characterized multiple T cell subtypes, including CD4⁺ naïve T cells, CD8⁺ effector memory T cells (Tem), CD8⁺ tissue-resident memory T cells (Trm), CD8⁺ exhausted T cells (Tex), regulatory T cells (Tregs), and cycling T cells. Myeloid cell subtypes were also delineated, comprising Langerhans cells, macrophages, mast cells, natural killer (NK) cells, conventional dendritic cells type 1 (cDC1) and type 2 (cDC2), as well as other skin cell populations (Fig. 6A). This cellular composition closely resembles that reported in previous studies [52]. Notably, the expression of key genes associated with the previously reported SFRP2⁺ fibroblast population—SFRP2, IL34, and CCL19—was predominantly localized within the fibroblast cluster (Fig. 6B). This spatial enrichment suggests that these fibroblasts may exist in a pro-inflammatory state, consistent with the characteristics described in the source datasets.

Regarding cell proportions, consistent with numerous previous reports, our huSA analysis revealed a decreased percentage of Langerhans cells in psoriatic skin lesions (Fig. 6C). Prior studies have highlighted increased intercellular communication between fibroblasts and keratinocytes. In the present analysis, huSA not only recapitulated these robust interactions but also delineated specific interaction subtypes with greater granularity. Notably, pronounced signaling was observed between COL23A1**⁺** fibroblasts and basal keratinocytes (Fig. 6D), suggesting a potentially specialized role of this fibroblast subset in epithelial–mesenchymal crosstalk. Furthermore, previous studies have reported cell type–specific communication mediated by multiple ligand–receptor interactions, such as CCL19–CCR7 and CXCL14–CXCR4 between fibroblasts and myeloid cells. The huSA not only recapitulated these interactions but also resolved them at the subtype level. Specifically, interactions involving COL23A1⁺, CXCL12⁺, and CCN5⁺ fibroblast subtypes with cDC1 cells were identified (Fig. 6E), suggesting a potential role for these fibroblast subsets in the recruitment and modulation of dendritic cell populations. Collectively, these findings demonstrate that the huSA-based workflow can reliably reproduce known cellular interactions while also uncovering novel insights into the pathophysiology of skin diseases.

### Personalized analysis of datasets from ‘Integration’ reveals distinct fibroblast subsets potentially contributing to inflammation in PN

**Figure 7:**
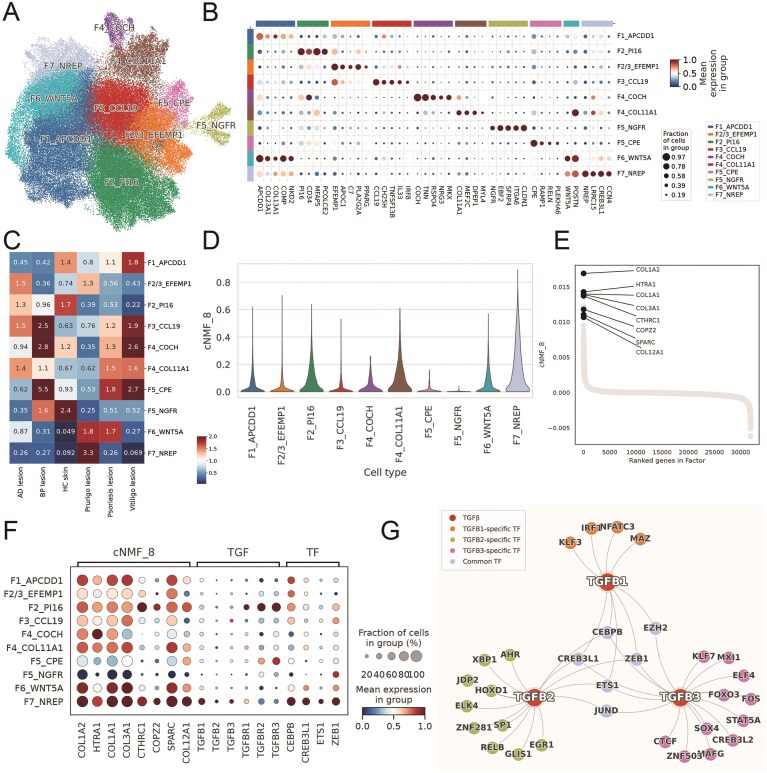
A profibrotic F7_NREP fibroblast subpopulation is enriched in prurigo nodularis (PN). A. UMAP projection of 95 753 integrated skin fibroblasts, identifying 10 distinct subsets. B. Dot plot showing marker gene expression defining fibroblast subsets. C. Heatmap of Ro/e analysis showing relative fibroblast subset abundance across skin diseases. D. Violin plot of consensus non-negative matrix factorization (cNMF) program 8 (Profibrotic Program) activity across fibroblast subsets in PN. E. Gene ranking within the cNMF_8. F. Dot plot of mean expression for cNMF8 program genes, TGFB isoforms, and co-regulating transcription factors across fibroblast subsets. G. Network plot shows SCENIC-inferred regulatory relationship.

To demonstrate the practical applications of the integrated data within the huSA database, we conducted an in-depth analysis utilizing the merged datasets of ‘Integration’ module. Specifically, we extracted fibroblast profiles from multiple skin conditions—including atopic dermatitis (AD), psoriasis, prurigo nodularis (PN), vitiligo, bullous pemphigoid (BP), and healthy controls (Supplementary Table 2)—with the aim of further characterizing fibroblast heterogeneity across distinct dermatological diseases. The integrated fibroblast atlas comprised a total of 95 753 fibroblasts, with 31.1% derived from normal skin samples, 26.2% from PN samples, 20.4% from AD samples, 19.4% from psoriasis samples, 1.8% from vitiligo samples, and 1% from BP samples (Supplementary Fig. 1A). Following the comprehensive fibroblast classification scheme approach outlined by Steele et al. [53], our single-cell analysis delineated 10 fibroblast subsets with differential expression patterns: F1_APCDD1 (APC down-regulated 1), F2_PI16 (Peptidase Inhibitor 16), F2/3_EFEMP1 (EGF Containing Fibulin Extracellular Matrix Protein 1), F3_CCL19 (C-C Motif Chemokine Ligand 19), F4_COCH (Cochlin), F4_COL11A1 (Collagen Type XI Alpha 1 Chain), F5_CPE (Carboxypeptidase E), F5_NGFR (Nerve Growth Factor Receptor), F6_WNT5A (Wnt Family Member 5A), F7_NREP (Neuronal Regeneration Related Protein) (Fig. 7A-B). The F7_NREP, F6_WNT5A, and F2/3_EFEMP1 fibroblast subsets were enriched, while F7_NREP showed specific enrichment exclusively in PN (Fig. 7C, Supplementary Fig. 1B). And the profile of F7_NREP were distinct through comparing with the expression patterns of established fibroblast markers (Supplementary Fig. 1C).

**Figure 8:**
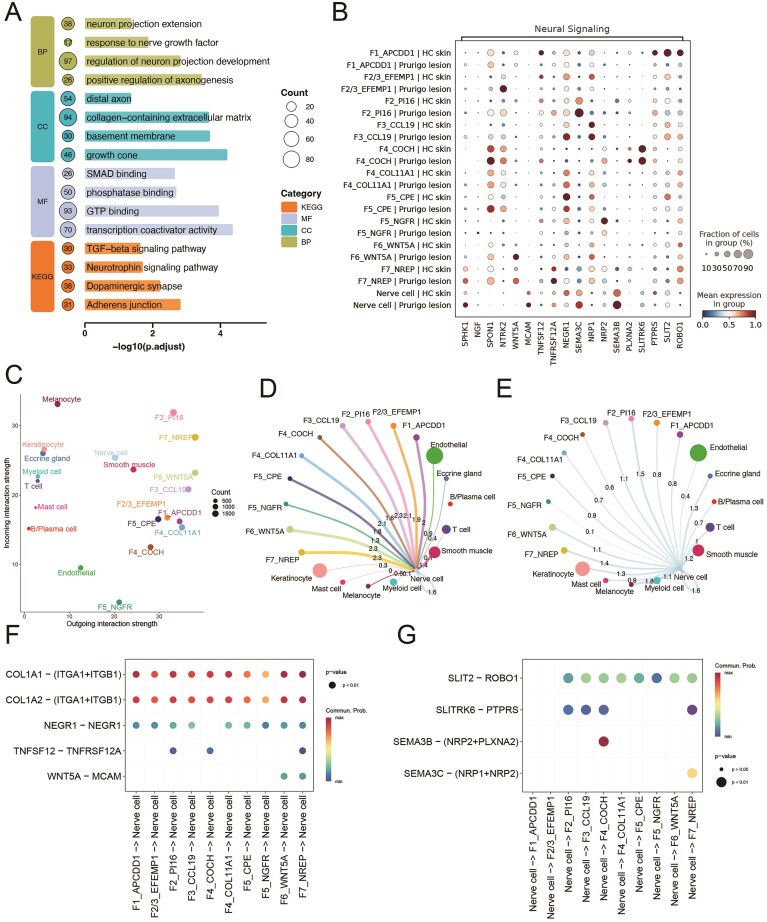
Functional characteristics and intercellular communication of fibroblast subpopulations in Prurigo Nodularis (PN). A. The diagram shows pathway enrichment analysis of the F7_NREP fibroblast subpopulation. B. Dot plot shows the neural signaling related genes expression in fibroblast subpopulations and nerve cells from PN and healthy skin. C. The dotplot displays the incoming and outgoing interaction strength for each cell type. D–E. Network plot of incoming signals to nerve cells(D) and outgoing signaling from nerve cells(E). F–G. The dotplot shows predicted ligand-receptor interactions from fibroblast clusters to nerve cells(F) and nerve cells to fibroblast subtypes(G).

To elucidate the function of F7_NREP fibroblasts, we performed consensus non-negative matrix factorization (cNMF) analysis (Supplementary Table 3). Among the identified programs, one (cNMF8) was predominantly activated in F7_NREP, whereas its activation was weaker or absent in other cell types (Fig. 7D, Supplementary Fig. 1D). The cNMF program 8 (cNMF8), which we named as ‘Profibrotic Program’, involves genes pivotal for ECM synthesis, including COL1A2, HTRA1, COL1A1, and COL3A1 (Fig. 7E-F, Supplementary Fig. 1E), underscoring their specialized capacity for robust ECM deposition [54]. Critically, this heightened fibrogenic state in F7_NREP cells were highly expressed of potent pro-fibrotic cytokines TGFB1, TGFB2, and TGFB3 [55], suggesting it is a key driver of the dermal fibrosis characteristic of PN lesions. The SCENIC analysis identified four transcription factors—CEBPB, EZH2, CREB3L1, and ETS1—that are predicted to co-regulate all three TGFB isoforms (Fig. 7G). Notably, these transcription factors exhibited higher expression in F7_NREP fibroblasts compared to other fibroblast subsets (Fig. 7F). Consistent with our cNMF analysis, pathway enrichment analysis revealed significant involvement of ECM-related processes, including the TGF-beta signaling pathway, collagen-containing extracellular matrix, and others (Fig. 8A). These findings underscore its critical involvement in ECM remodeling and fibrosis, which are established hallmarks of PN’s chronic fibrotic lesions [56]. Intriguingly, beyond ECM remodeling, F7_NREP subpopulation demonstrated striking enrichment in several neuro-related pathways such as neurotrophin signaling pathway, positive regulation of axonogenesis, neuron projection extension and regulation of neuron projection development (Fig. 8A). This suggests that F7_NREP are not merely structural cells but actively contribute to the neurogenic inflammation characteristic of NP, potentially by promoting neuronal sensitization and aberrant nerve fiber sprouting.

SPHK1 can catalyze the formation of sphingosine-1-phosphate (S1P), a potent bioactive lipid mediator that directly activates sensory neurons and enhances pruriceptive signaling [57,58]. Notably, the SPHK1 gene was found to be highly expressed in the F7_NREP fibroblast subpopulation (Fig. 8B, Supplementary Fig. 2A), suggesting a potential role for this subpopulation in promoting neuronal sensitization and sustaining the chronic itch cycle characteristic of PN [59]. we further performed intercellular communication analysis, which revealed that the F7_NREP subpopulation exhibited the highest outgoing interaction strength in PN lesional skin (Fig. 8C). Analysis of incoming signals to Nerve cells revealed that F2_PI16, F6_WNT5A, and F7_NREP cell types were the most prominent senders, each exhibiting a maximal interaction strength of 2.3 (Fig. 8D). Conversely, when examining outgoing signals from Nerve cells, F2_PI16 cells and F7_NREP cells were identified as the primary target populations (Fig. 8E). Notably, robust signaling via collagen I (COL1A1/COL1A2), originating from multiple fibroblast-like clusters to its integrin receptor (ITGA1/ITGB1) on nerve cells was represented (Fig. 8F, Supplementary Fig. 2B), suggesting substantial modulation of the neuronal microenvironment through extracellular matrix (ECM) interactions. Furthermore, NEGR1-NEGR1 homophilic interactions, crucial for neuronal adhesion and growth [60,61], were observed between several stromal clusters and nerve cells. Pro-inflammatory and tissue-remodeling signals, exemplified by the TNFSF12 (TWEAK)-TNFRSF12A (Fn14) axis [62], were predicted to predominantly emanate from F2/3_EFEMP1, F4_COL11A1, and particularly F7_NREP clusters, targeting nerve cells. WNT5A-MCAM signaling, implicated in cell migration and inflammation [63], was prominently predicted from F6_WNT5A and F7_NREP clusters to nerve cells. The SLIT2-ROBO1 pathway, a canonical axon repulsion and cell migration inhibitory system [64], is predicted between nerve cells and multiple fibroblast subtypes(Fig. 8G, Supplementary Fig. 2C). These suggest that fibroblast subsets may actively orchestrate a microenvironment conducive to neuronal sensitization and aberrant nerve growth, thereby perpetuating the hallmark pruritus and neuroinflammation of the disease.

## Discussion

The skin research has been undergoing transformative progress with the rapid evolution of high-throughput sequencing technologies. To fully harness the potential of these datasets, it is crucial to develop a skin diseases database that prioritizes unified data collection, standardization, curation, integration, and visualization. Thus, we developed huSA, a comprehensive platform that combines detailed cell annotations with robust analytical capabilities, empowering researchers to explore, integrate, and reanalyze data with precision. The database stands out not only for its meticulous curation but also for its user-centric design, facilitating the exploration of complex datasets across a wide spectrum of skin diseases.

The huSA is distinguished by its defining features, which collectively enhance its value as a specialized resource for skin research. Through these innovations, huSA aims to bridge the gap between raw data and actionable insights, offering a foundational tool to advance the understanding of skin biology. Firstly, the huSA is the most extensive database dedicated to skin research up to now, encompassing approximately 4.6 million single cells. These data are derived from 63 high-quality datasets covering 17 different skin diseases, providing a robust foundation for a wide range of analyses and comparisons. Secondly, huSA employs a standardized pipeline for the analysis of multiple datasets, ensuring the reliability, consistency, and reproducibility of the results. This analytical framework enables robust cross-dataset comparisons, significantly enhancing the overall quality of the database. Thirdly, huSA provides comprehensive suite of tools for comparative analysis across various skin diseased involved tissues/organs, datasets, and cell types. By the way, huSA is equipped with intuitive and user-friendly visualization interfaces, such as the ‘cell × gene’ and ‘Cirrocumulus’ platforms, allowing users to customize analyses based on their interests and catering to diverse research needs. While other databases lack integrated disease-specific data, limiting their analytical power and result universality, huSA provides integrated data and downstream analysis results from the same disease in the ‘Integration’ module, enabling users to derive more reliable biological discoveries based on a larger sample size.

To stay current with the fast-paced advancements in the field, huSA will undergo annual updates incorporate newly available datasets. We remain dedicated to maintaining and enhancing its functionality to meet the evolving needs of researchers. Currently, we are actively developing an upgraded platform designed to continuously gather more datasets of other skin diseases and integrate the latest single-cell transcriptomic data, genomic data, epigenetic data, and other single-cell omics data such as scATAC-seq, scTCR-seq, scBCR-seq, and CITE-seq. This enhanced platform will empower users to upload their own data and perform relevant analyses using the resources of this database, such as cell annotation, cell similarity analysis, and bulk data deconvolution, further enhancing the database’s utility and user experience. Through ongoing improvements and expansions, huSA aims to remain a cutting-edge resource, supporting advancements in skin research and accelerating the discovery of novel biological insights. In summary, huSA is a comprehensive resource that fully analyzes bulk RNA-seq, scRNA-seq, and spatial transcriptomic datasets from 17 skin diseases, supporting the global skin disease research community and extending its applications beyond.

## Supplementary Material

baag009_Supplemental_Files

## Data Availability

The huSA 1.0 is available online (https://humanskinatlas.com/index.html).

## References

[1] Harris-Tryon TA, Grice EA. Microbiota and maintenance of skin barrier function. Science. 2022;376:940–45. 10.1126/science.abo0693 35617415

[2] Grice EA, Segre JA. The skin microbiome. Nat Rev Micro. 2011;9:244–53. 10.1038/nrmicro2537 PMC3535073 21407241

[3] Ávalos-Díaz E, Esparza RH. Dermatological autoimmune diseases. In: Autoimmunity: From Bench to Bedside [Internet]: El Rosario University Press. 2013; 29087650

[4] Theocharidis G, Tekkela S, Veves A et al. Single-cell transcriptomics in human skin research: available technologies, technical considerations and disease applications. Exp. Dermatol. 2022;31:655–73. 10.1111/exd.14547 35196402 PMC9311140

[5] Karimkhani C, Dellavalle RP, Coffeng LE et al. Global skin disease morbidity and mortality: an update from the global burden of disease study 2013. JAMA Dermatol. 2017;153:406–12. 10.1001/jamadermatol.2016.5538 28249066 PMC5817488

[6] Hay RJ, Johns NE, Williams HC et al. The global burden of skin disease in 2010: an analysis of the prevalence and impact of skin conditions. J. Invest. Dermatol. 2014;134:1527–34. 10.1038/jid.2013.446 24166134

[7] Global incidence, prevalence, years lived with disability (YLDs), disability-adjusted life-years (DALYs), and healthy life expectancy (HALE) for 371 diseases and injuries in 204 countries and territories and 811 subnational locations, 1990–2021: a systematic analysis for the Global Burden of Disease Study 2021. Lancet. 2024;403:2133–61. 38642570 10.1016/S0140-6736(24)00757-8 PMC11122111

[8] Kolkhir P, Giménez-Arnau AM, Kulthanan K et al. Urticaria. Nat Rev Dis Primers. 2022;8:61. 10.1038/s41572-022-00389-z 36109590

[9] Scheinman PL, Vocanson M, Thyssen JP et al. Contact dermatitis. Nat Rev Dis Primers. 2021;7:38. 10.1038/s41572-021-00271-4 34045488

[10] Langan SM, Irvine AD, Weidinger S., dermatitis Atopic. Lancet. 2020;396:345–60. 10.1016/S0140-6736(20)31286-1 32738956

[11] Boehncke W-H, Schön MP. Psoriasis. Lancet (London, England). 2015;386:983–94. 10.1016/S0140-6736(14)61909-7 26025581

[12] Griffiths CEM, Armstrong AW, Gudjonsson JE et al. Psoriasis. Lancet. 2021;397:1301–15. 10.1016/S0140-6736(20)32549-6 33812489

[13] Hoi A, Igel T, Mok CC et al. Systemic lupus erythematosus. Lancet. 2024;403:2326–38. 10.1016/S0140-6736(24)00398-2 38642569

[14] Volkmann ER, Andréasson K, Smith V. Systemic sclerosis. Lancet. 2023;401:304–18. 10.1016/S0140-6736(22)01692-0 36442487 PMC9892343

[15] Frisoli ML, Essien K, Harris JE. Vitiligo: mechanisms of pathogenesis and treatment. Annu. Rev. Immunol. 2020;38:621–48. 10.1146/annurev-immunol-100919-023531 32017656

[16] Schmidt E, Kasperkiewicz M, Joly P. Pemphigus. Lancet. 2019;394:882–94. 10.1016/S0140-6736(19)31778-7 31498102

[17] Davidson A, Diamond B. Autoimmune diseases. N Engl J Med. 2001;345:340–50. 10.1056/NEJM200108023450506 11484692

[18] Yu Y, Dunaway S, Champer J et al. Changing our microbiome: probiotics in dermatology. Br J Dermatol. 2020;182:39–46. 10.1111/bjd.18659 31049923

[19] Moro F, Fania L, Sinagra JLM et al. Bullous pemphigoid: trigger and predisposing factors. Biomolecules. 2020;10. 10.3390/biom10101432 PMC7600534 33050407

[20] Bieber T . Atopic dermatitis: an expanding therapeutic pipeline for a complex disease. Nat Rev Drug Discovery. 2022;21:21–40. 10.1038/s41573-021-00266-6 34417579 PMC8377708

[21] Siegel CH, Sammaritano LR. Systemic lupus erythematosus: a review. JAMA. 2024;331:1480–91. 10.1001/jama.2024.2315 38587826

[22] FitzGerald O, Ogdie A, Chandran V et al. Psoriatic arthritis. Nat Rev Dis Primers. 2021;7:59. 10.1038/s41572-021-00293-y 34385474

[23] Shi M, Zhang H, Chen X et al. Clinical features of atopic dermatitis in a hospital-based setting in China. Journal of the European Academy of Dermatology and Venereology: JEADV. 2011;25:1206–12. 10.1111/j.1468-3083.2010.03953.x 21214635

[24] Kiriakidou M, Ching CL. Systemic lupus erythematosus. Ann. Intern. Med. 2020;172:ITC81–96. 10.7326/AITC202006020 32479157

[25] Jung SM, Kim W-U. Targeted immunotherapy for autoimmune disease. Immune Network. 2022;22:e9. 10.4110/in.2022.22.e9 35291650 PMC8901705

[26] Fugger L, Jensen LT, Rossjohn J. Challenges, progress, and prospects of developing therapies to treat autoimmune diseases. Cell. 2020;181:63–80. 10.1016/j.cell.2020.03.007 32243797

[27] Tang F, Barbacioru C, Wang Y et al. mRNA-Seq whole-transcriptome analysis of a single cell. Nat. Methods. 2009;6:377–82. 10.1038/nmeth.1315 19349980

[28] Billi AC, Ma F, Plazyo O et al. Nonlesional lupus skin contributes to inflammatory education of myeloid cells and primes for cutaneous inflammation. Sci. Transl. Med. 2022;14:eabn2263. 10.1126/scitranslmed.abn2263 35476593 PMC9169615

[29] Zheng M, Hu Z, Mei X et al. Single-cell sequencing shows cellular heterogeneity of cutaneous lesions in lupus erythematosus. Nat Commun. 2022;13:7489. 10.1038/s41467-022-35209-1 36470882 PMC9722937

[30] Goto M, Takahashi H, Yoshida R et al. Age-associated CD4(+) T cells with B cell-promoting functions are regulated by ZEB2 in autoimmunity. Sci Immunol. 2024;9:eadk1643. 10.1126/sciimmunol.adk1643 38330141

[31] He H, Suryawanshi H, Morozov P et al. Single-cell transcriptome analysis of human skin identifies novel fibroblast subpopulation and enrichment of immune subsets in atopic dermatitis. J. Allergy Clin. Immunol. 2020; 10.1016/j.jaci.2020.01.042 32035984

[32] Ma F, Plazyo O, Billi AC et al. Single cell and spatial sequencing define processes by which keratinocytes and fibroblasts amplify inflammatory responses in psoriasis. Nat Commun. 2023;14:3455. 10.1038/s41467-023-39020-4 37308489 PMC10261041

[33] Zeng J, Zhang Y, Shang Y et al. CancerSCEM: a database of single-cell expression map across various human cancers. Nucleic Acids Res. 2022;50: D1147-D55. 10.1093/nar/gkab905 PMC8728207 34643725

[34] Deng Y, Chen P, Xiao J et al. SCAR: single-cell and spatially-resolved cancer resources. Nucleic Acids Res. 2024;52:D1407–D17. 10.1093/nar/gkad753 37739405 PMC10767865

[35] Gao X, Hong F, Hu Z et al. ABC portal: a single-cell database and web server for blood cells. Nucleic Acids Res. 2023;51:D792–804. 10.1093/nar/gkac646 35920330 PMC9825444

[36] Zheng GXY, Terry JM, Belgrader P et al. Massively parallel digital transcriptional profiling of single cells. #N/A. 2017;8:14049. 10.1038/ncomms14049 PMC5241818 28091601

[37] Wolock SL, Lopez R, Klein AM. Scrublet: computational identification of cell doublets in single-cell transcriptomic data. Cell Syst. 2019;8: 281–91.e9. 10.1016/j.cels.2018.11.005 PMC6625319 30954476

[38] Virshup I, Bredikhin D, Heumos L et al. The scverse project provides a computational ecosystem for single-cell omics data analysis. Nat. Biotechnol. 2023;41:604–606. 10.1038/s41587-023-01733-8 37037904

[39] Dominguez Conde C, Xu C, Jarvis LB et al. Cross-tissue immune cell analysis reveals tissue-specific features in humans. Science. 2022;376:eabl5197. 10.1126/science.abl5197 35549406 PMC7612735

[40] Yang L, Ng YE, Sun H et al. Single-cell Mayo Map (scMayoMap): an easy-to-use tool for cell type annotation in single-cell RNA-sequencing data analysis. BMC Biol. 2023;21. 10.1186/s12915-023-01728-6 PMC10588107 37858214

[41] Van de Sande B, Flerin C, Davie K et al. A scalable SCENIC workflow for single-cell gene regulatory network analysis. Nat. Protoc. 2020;15:2247–76. 10.1038/s41596-020-0336-2 32561888

[42] Efremova M, Vento-Tormo M, Teichmann SA et al. CellPhoneDB: inferring cell–cell communication from combined expression of multi-subunit ligand–receptor complexes. Nat. Protoc. 2020;15:1484–506. 10.1038/s41596-020-0292-x 32103204

[43] Zeng Z, Ma Y, Hu L et al. OmicVerse: a framework for bridging and deepening insights across bulk and single-cell sequencing. Nat Commun. 2024;15:5983. 10.1038/s41467-024-50194-3 39013860 PMC11252408

[44] Kang M, Armenteros JJA, Gulati GS et al. Mapping single-cell developmental potential in health and disease with interpretable deep learning. In: bioRxiv. 2024;

[45] Alghamdi N, Chang W, Dang P et al. A graph neural network model to estimate cell-wise metabolic flux using single-cell RNA-seq data. Genome Res. 2021;31:1867–84. 10.1101/gr.271205.120 34301623 PMC8494226

[46] Blondel VD, Guillaume J-L, Lambiotte R et al. Fast unfolding of communities in large networks. J Stat Mech: Theory Exp. 2008;2008:P10008. 10.1088/1742-5468/2008/10/P10008

[47] Long Y, Ang KS, Li M et al. Spatially informed clustering, integration, and deconvolution of spatial transcriptomics with GraphST. Nat Commun. 2023;14:1155. 10.1038/s41467-023-36796-3 36859400 PMC9977836

[48] Traag VA, Waltman L, van Eck NJ. From louvain to leiden: guaranteeing well-connected communities. Sci Rep. 2019;9:5233. 10.1038/s41598-019-41695-z 30914743 PMC6435756

[49] Guo X, Zhang Y, Zheng L et al. Global characterization of T cells in non-small-cell lung cancer by single-cell sequencing. Nat. Med. 2018;24:978–85. 10.1038/s41591-018-0045-3 29942094

[50] Heumos L, Ji Y, May L et al. Pertpy: an end-to-end framework for perturbation analysis. bioRxi. 2024: 2024.08.04.606516. 10.1038/s41592-025-02909-7 PMC12904789 41476114

[51] Kotliar D, Veres A, Nagy MA et al. Identifying gene expression programs of cell-type identity and cellular activity with single-cell RNA-Seq. Elife. 2019;8. 10.7554/eLife.43803 PMC6639075 31282856

[52] Ma F, Plazyo O, Billi AC et al. Single cell and spatial sequencing define processes by which keratinocytes and fibroblasts amplify inflammatory responses in psoriasis. Nat Commun. 2023;14. 10.1038/s41467-023-39020-4 PMC10261041 37308489

[53] Steele L, Admane C, Chakala KP et al. A single cell and spatial genomics atlas of human skin fibroblasts in health and disease. bioRxi. 2024: 2024.12.23.629194. 10.1038/s41590-025-02267-8 PMC12479362 40993240

[54] Naba A . Mechanisms of assembly and remodelling of the extracellular matrix. Nat Rev Mol Cell Biol. 2024;25:865–85. 10.1038/s41580-024-00767-3 39223427 PMC11931590

[55] Akhurst RJ, Hata A. Targeting the TGFβ signalling pathway in disease. Nat Rev Drug Discov. 2012;11:790–811. 10.1038/nrd3810 23000686 PMC3520610

[56] Lu J, Liu Q, Wang L et al. Increased expression of latent TGF-β-binding protein 4 affects the fibrotic process in scleroderma by TGF-β/SMAD signaling. Lab. Invest. 2017;97:591–601. 10.1038/labinvest.2017.20 28263294

[57] Welch SP, Sim-Selley LJ, Selley DE. Sphingosine-1-phosphate receptors as emerging targets for treatment of pain. Biochem. Pharmacol. 2012;84:1551–62. 10.1016/j.bcp.2012.08.010 22971335

[58] Terao R, Honjo M, Ueta T et al. Light stress-induced increase of sphingosine 1-phosphate in photoreceptors and its relevance to retinal degeneration. Int J Mol Sci. 2019;20. 10.3390/ijms20153670 PMC6696268 31357484

[59] Gray N, Limberg MM, Bräuer AU et al. Novel functions of S1P in chronic itchy and inflammatory skin diseases. J. Eur. Acad. Dermatol. Venereol. 2022;36:365–72. 10.1111/jdv.17764 34679239

[60] Singh K, Loreth D, Pöttker B et al. Neuronal growth and behavioral alterations in mice deficient for the psychiatric disease-associated negr1 gene. Front. Mol. Neurosci. 2018;11:30. 10.3389/fnmol.2018.00030 29479305 PMC5811522

[61] Poplawski GHD, Lie R, Hunt M et al. Adult rat myelin enhances axonal outgrowth from neural stem cells. Sci. Transl. Med. 2018;10. 10.1126/scitranslmed.aal2563 PMC8377986 29794059

[62] Liu Q, Xiao S, Xia Y. TWEAK/Fn14 activation participates in skin inflammation. Mediat Inflamm. 2017; 2017; 6746870. 10.1155/2017/6746870 PMC5606047 29038621

[63] Wang Z, Xu Q, Zhang N et al. CD146, from a melanoma cell adhesion molecule to a signaling receptor. Signal Transduct Target Ther. 2020;5:148. 10.1038/s41392-020-00259-8 32782280 PMC7421905

[64] Lowery LA, Van Vactor D. The trip of the tip: understanding the growth cone machinery. Nat Rev Mol Cell Biol. 2009;10:332–43. 10.1038/nrm2679 19373241 PMC2714171

