# Supplementary material for: huSA: a comprehensive database for multi-dimensional resolution of bulk, single cell and spatial transcription profiles in skin diseases
Source: Database (Oxford). 2026 Feb 20;2026:baag009. doi: 10.1093/database/baag009 (PMC12923168; doi:10.1093/database/baag009)
Supplement: baag009_Supplemental_Files [file baag009_supplemental_files.zip › Supplementary table 1.docx]

Supplementary table 1. Statistics of huSA

| Skin status | Tissues/Organs | Sample counts | | |
| --- | --- | --- | --- | --- |
|  |  | scRNA-seq | Bulk RNA-seq | Spatial |
| Healthy control (HC) | Skin, Blood | 450 | 433 | / |
| Psoriasis | Skin, Blood, Synovial fluid, Synovial tissue | 437 | 80 | 44 |
| Atopic dermatitis (AD) | Skin, Blood | 82 | 226 | 19 |
| Dermatomyositis | Blood | 20 | 88 | / |
| Behçet’s disease (BD) | Blood | 8 | 10 | / |
| Systemic lupus erythematosus (SLE) | Skin, Blood, Kidney | 283 | 309 | / |
| Discoid lupus erythematosus (DLE) | Skin | 10 | / | / |
| Subacute cutaneous lupus erythematosus (SCLE) | Skin | 3 | / | / |
| Vitiligo | Skin, Blood | 27 | 4 | / |
| Lichen planus (LP) | Skin | 2 | 35 | / |
| Bullous pemphigoid (BP) | Skin, Blood, Blister | 18 | / | / |
| Prurigo nodularis (PN) | Skin | 24 | / | / |
| Clinical indeterminate rashes (CIRs) | Skin | 6 | / | / |
| Chronic nodular prurigo (CNPG) | Skin | 7 | / | / |
| Hidradenitis suppurativa (HS) | Skin | 8 | / | / |
| Dupilumab-associated head and neck dermatitis (DAHND) | Skin | 6 | / | / |
| Systemic sclerosis (SSc) | Skin | 12 | 301 | / |
| Sjogren^,^s syndrome (SS) | Blood | 31 | 16 | / |
| Sum | / | 1434 | 1502 | 63 |
