# Supplementary figures and images for: huSA: a comprehensive database for multi-dimensional resolution of bulk, single cell and spatial transcription profiles in skin diseases
Source: Database (Oxford). 2026 Feb 20;2026:baag009. doi: 10.1093/database/baag009 (PMC12923168; doi:10.1093/database/baag009)

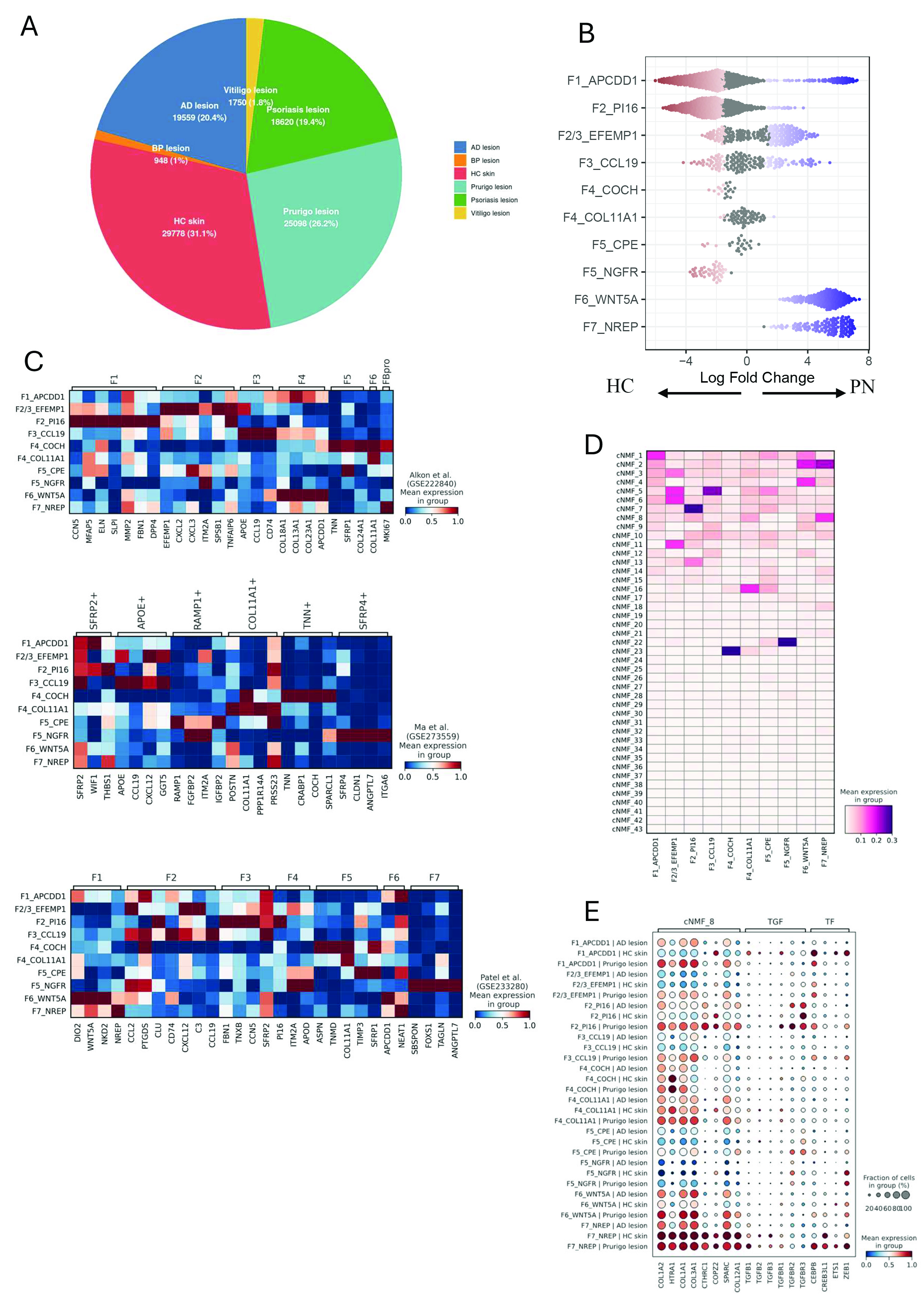

Supplement: baag009_Supplemental_Files [file baag009_supplemental_files.zip › Fig_S1.tiff]

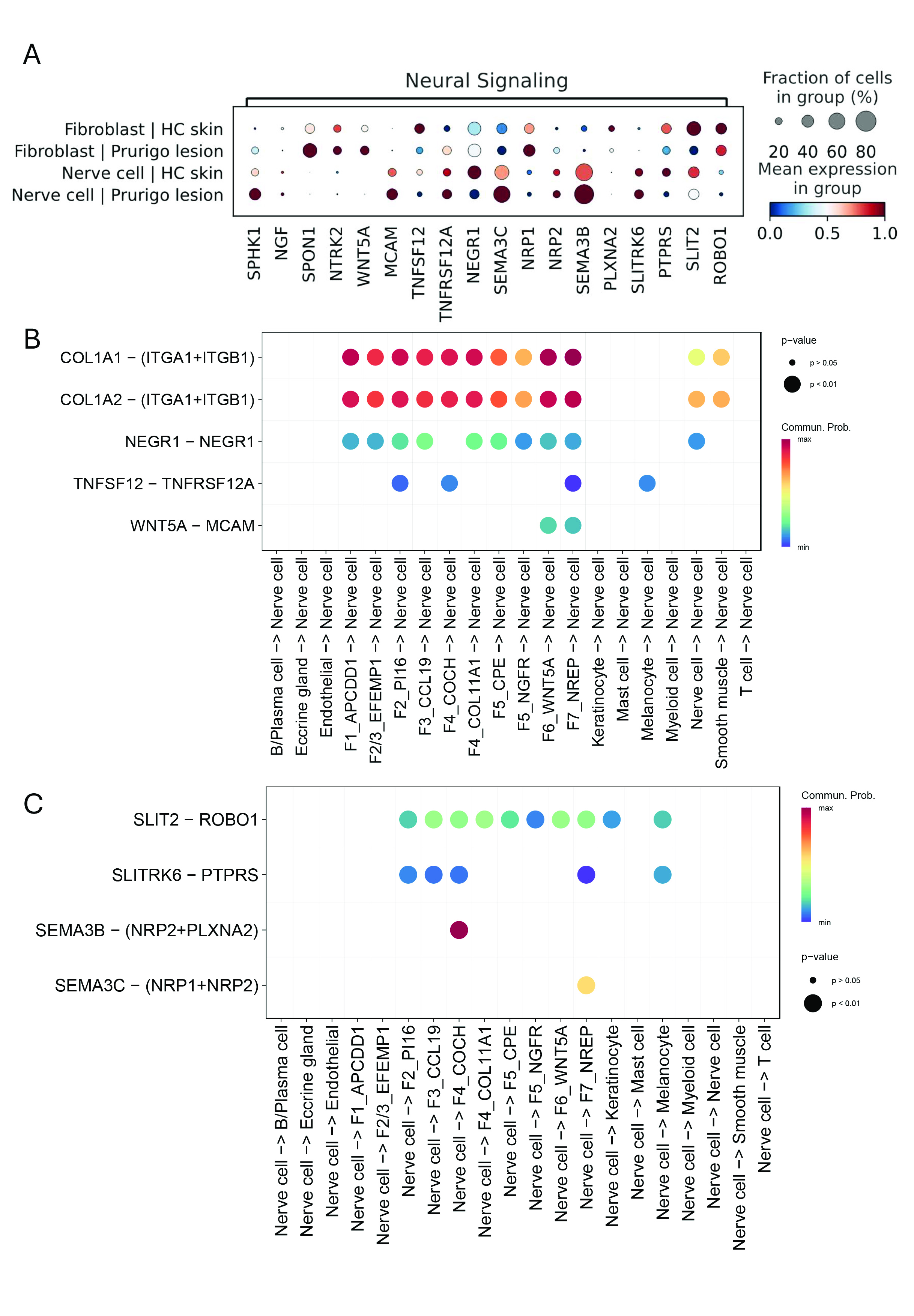

Supplement: baag009_Supplemental_Files [file baag009_supplemental_files.zip › Fig_S2.tiff]
